# Coexistence of ankylosing spondylitis and Behçet's disease: Successful treatment with upadacitinib

**DOI:** 10.1002/iid3.1242

**Published:** 2024-04-05

**Authors:** Krasimir Kraev, Petar Uchikov, Bozhidar Hristov, Maria Kraeva, Yordanka Basheva‐Kraeva, Stanislava Popova‐Belova, Milena Sandeva, Dzhevdet Chakarov, Snezhanka Dragusheva, Mariela Geneva‐Popova

**Affiliations:** ^1^ Department of Propaedeutics of Internal Diseases “Prof. Dr. Anton Mitov”, Faculty of Medicine Medical University of Plovdiv Plovdiv Bulgaria; ^2^ Department of Special Surgery, Faculty of Medicine Medical University of Plovdiv Plovdiv Bulgaria; ^3^ Second Department of Internal Diseases, Section “Gastroenterology”, Medical Faculty Medical University of Plovdiv Plovdiv Bulgaria; ^4^ Department of Otorhinolaryngology, Medical Faculty Medical University of Plovdiv Plovdiv Bulgaria; ^5^ Department of Ophthalmology, Medical Faculty Medical University of Plovdiv Plovdiv Bulgaria; ^6^ Department of Midwifery, Faculty of Public Health Medical University of Plovdiv Plovdiv Bulgaria; ^7^ Department of Propaedeutics of Surgical Diseases, Section of General Surgery, Faculty of Medicine Medical University of Plovdiv Plovdiv Bulgaria; ^8^ Department of Nursing Care, Faculty of Public Health Medical University of Plovdiv Plovdiv Bulgaria

**Keywords:** ankylosing spondylitis, Behçet's disease, case report, sacroiliac joints, ulcers

## Abstract

**Background:**

Ankylosing spondylitis (AS) and Behçet's disease (BD) are distinct inflammatory disorders, but their coexistence is a rare clinical entity. This case sheds light on managing this complex scenario with Janus kinase (JAK) inhibitors.

**Case Presentation:**

A 42‐year‐old woman presented with a decade‐long history of lower back pain, nocturnal spinal discomfort, recurrent eye issues, oral and genital ulcers, hearing loss, pus formation in the left eye, and abdominal pain. Multidisciplinary consultations and diagnostic tests confirmed AS (HLA‐B27 positivity and sacroiliitis) and BD (HLA‐B51). Elevated acute‐phase markers were observed.

**Conclusion:**

This case fulfills diagnostic criteria for both AS and BD, emphasizing their coexistence. Notably, treatment with upadacitinib exhibited promising efficacy, underscoring its potential as a therapeutic option in patients with contraindications for conventional treatments. Our findings illuminate the intricate management of patients presenting with these two diverse systemic conditions and advocate for further exploration of JAK inhibitors in similar cases.

## Graphical abstract

In this study, we present a case of a 42‐year‐old female patient who exhibited simultaneous symptoms fulfilling diagnostic criteria for both ankylosing spondylitis and Behçet's disease. Notably, treatment with Upadacitinib showed promising efficacy in managing the complex clinical manifestations of both conditions, suggesting a potential therapeutic avenue for similar cases in the future.

## 1 INTRODUCTION

Ankylosing spondylitis (AS) is classified among the inflammatory rheumatic diseases, and specifically, within the category of inflammatory spondyloarthropathies.^1^ This chronic joint ailment primarily manifests in the spinal region, with a characteristic feature known as “night pain,” which severely disrupts sleep and the overall well‐being of patients.^1^

In contrast, Behçet's disease (BD), a rare systemic condition belonging to the vasculitides group, mainly affects young men aged 20–40. Evidence suggests a strong association with HLA‐B5 (B51) carriage, and it predominantly impacts individuals of Jewish, Arab, and Turkish descent.^2^ This systemic vasculitis can involve arteries and veins, irrespective of their size, and often presents with joint involvement and distinct ocular problems, setting it apart from the clinical manifestations seen in AS.^3^

While the coexistence of AS and BD has been documented in various articles,^4^, ^5^, ^6^ our study contributes a unique perspective. We delve into an innovative treatment approach, exploring the use of Janus kinase (JAK) inhibitors, specifically upadacitinib. This therapeutic exploration gains particular relevance when conventional treatment options are deemed unsuitable. Our research aims to provide invaluable insights into the management of this intricate clinical scenario, building upon the foundation of previous studies.^7^

## 2 CASE PRESENTATION

### 2.1 Clinical findings

A 42‐year‐old female patient presented with a decade‐long history of chronic pain and stiffness localized to the lower back, characterized by nocturnal spinal pain. Notably, there was no involvement of peripheral joints, and there was no personal or family history of skin psoriasis. The patient additionally presented with chronic ocular issues, including visual blurring, redness, photophobia, and hypopion, which were managed with the use of local and systemic corticosteroids. In the last year, she experienced the sudden onset of oral and genital ulcerations, accompanied by hearing loss and the presence of pus in the left eye. Additionally, the patient complained of abdominal pain, although there was no associated diarrhea or blood in stools.

### 2.2 Consultations

#### 2.2.1 Ophthalmologist

Examination revealed typical changes consistent with Behcet syndrome's panuveitis in the affected eye, including the presence of hypopyon. No signs of retinal damage were observed.

#### 2.2.2 Otorhinolaryngologist

The hearing loss was attributed to BD, as it coincided with the onset of other disease‐related symptoms.

#### 2.2.3 Gastroenterologist and abdominal surgeon

Comprehensive evaluations yielded no evidence of inflammatory diseases affecting the gastrointestinal tract, nor were there any signs of acute abdominal problems or other identifiable causes of abdominal pain. There was no indication of imminent complications such as Budd–Chiari syndrome.

### 2.3 Laboratory and radiographic studies

**Figure 1:**
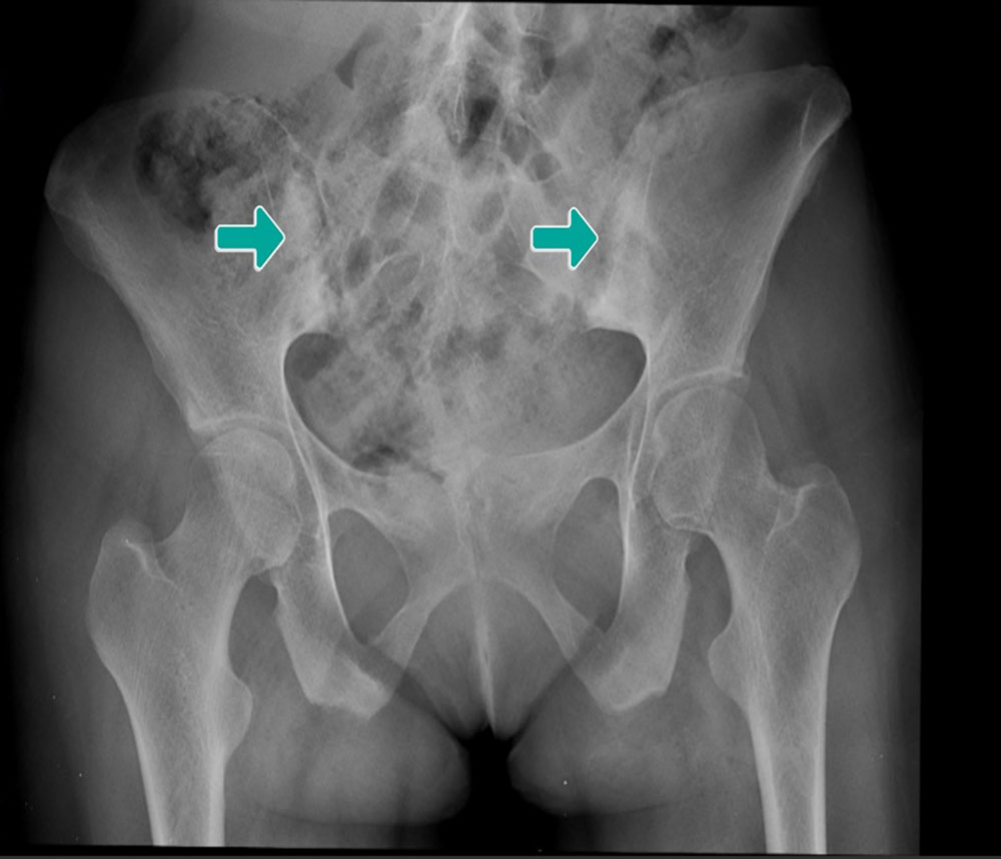
X‐ray with bilateral sacroiliitis.

**Figure 2:**
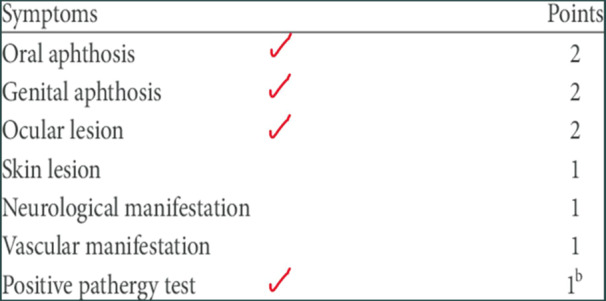
Patient's criteria for Behçet's disease.

**Figure 3:**
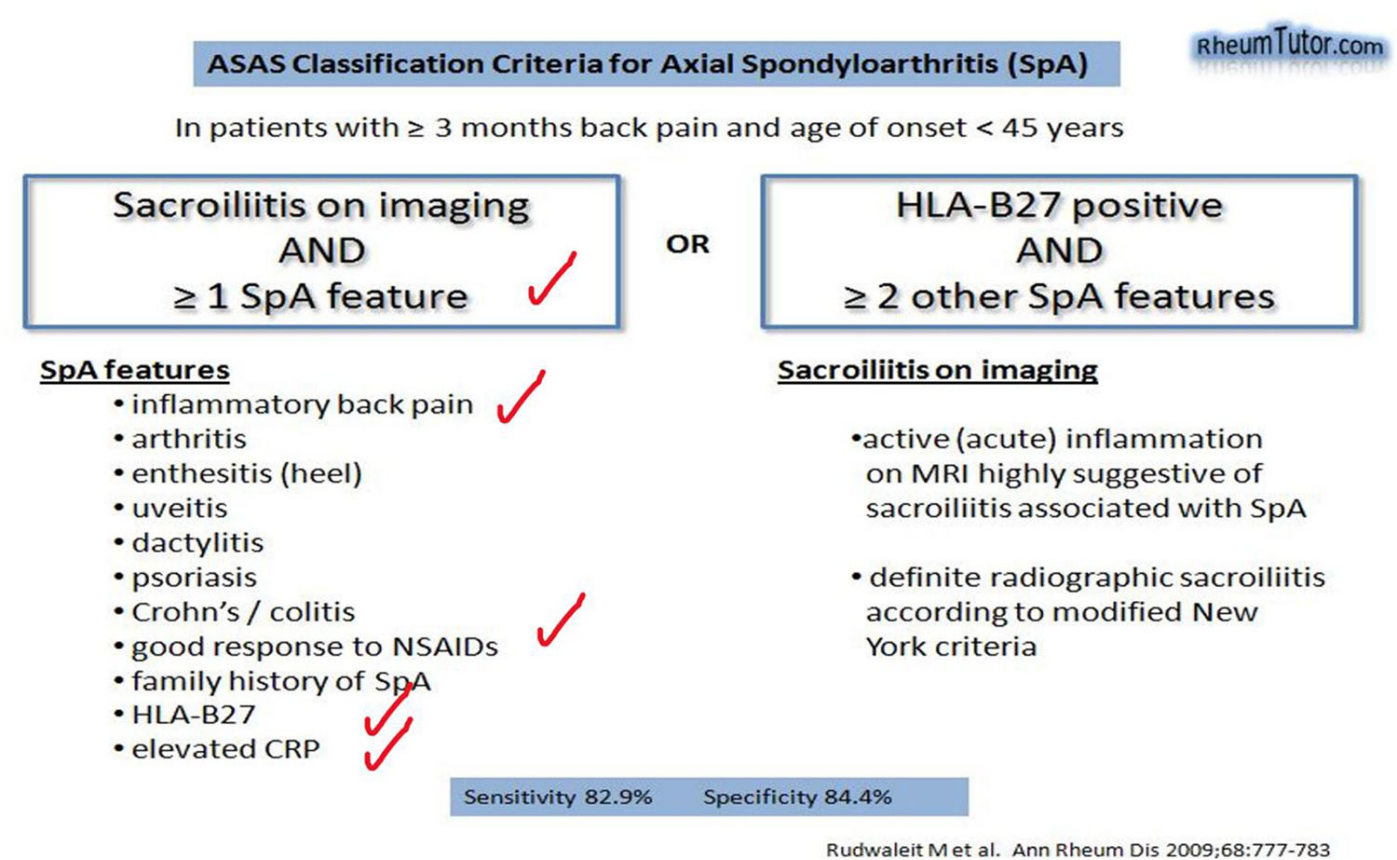
Patient's criteria for SpA (AS). AS, ankylosing spondylitis.

Laboratory assessments revealed a normal Complete Blood Count but elevated acute phase indicators, including an increased erythrocyte sedimentation rate and C‐reactive protein levels. HLA sampling confirmed the presence of HLA‐B27 and HLA‐B51 (B5) markers. Radiographic imaging displayed bilateral sacroiliitis graded at 2‐3, indicating the involvement of both sacroiliac joints (Figures 1, 2, 3).

### 2.4 Diagnostic assessment

Upon compiling the laboratory results, X‐ray findings, and the patient's medical history, a comprehensive assessment was performed to confirm the patient's adherence to the current classification criteria for both AS and BD.

### 2.5 Therapeutic interventions

Treatment was initiated with upadacitinib at a daily dosage of 15 mg. This choice was influenced by the reported efficacy of upadacitinib in AS and the existing studies supporting the use of JAK inhibitors in patients with refractory BD.^7^

Upon the first follow‐up, conducted 1 month subsequent to the initiation of treatment, the patient reported noteworthy alleviation in joint discomfort and mucosal ulcerations, encompassing both oral and genital regions. A subsequent in‐depth evaluation performed at the 3‐month mark revealed a remarkable amelioration in the majority of symptoms associated with both disease entities, with many presenting signs having undergone substantial improvement or achieving complete resolution. These findings underscore the potential efficacy and positive therapeutic outcomes of the prescribed treatment regimen in addressing the multifaceted manifestations of the patient's condition.

## 3 DISCUSSION

AS and BD are distinct entities within the realm of rheumatic diseases, each presenting a unique clinical spectrum and diagnostic challenges. Understanding the differential diagnosis, clinical manifestations, and therapeutic options is crucial for optimal patient management.

### 3.1 Clinical spectrum of BD

BD is renowned for its multisystem involvement, making it a complex diagnostic puzzle. One of the hallmark features of BD is ocular involvement, occurring in over 50% of affected individuals. Patients may experience chronic, recurrent uveitis, which can manifest as both anterior and posterior uveitis, often culminating in hypopyon formation. In severe cases, retinal damage can lead to vision loss.^8^

Aphthosis is perhaps the most characteristic symptom, with oral aphthosis affecting more than 90% of patients. Importantly, these oral ulcers typically heal without scarring or cicatrization. Genital aphthosis, found in over 80% of patients, contrasts by leading to the development of scars and cicatrices.^9^

Cutaneous symptoms, including erythema nodosum, are frequent, with the pathergy test serving as a diagnostic indicator. Arthritic complaints are present in approximately half of all BD cases. Typically, joint involvement presents as asymmetric oligoarthritis, primarily affecting peripheral joints, while sparing the axial skeleton. In rare instances, polyarticular involvement and the inclusion of sacroiliac joints and spinal segments may occur.^9^

Less common but potentially severe complications involve the nervous system, gastrointestinal tract, and the heart. Among these, the development of Bud‐Chiari syndrome stands as one of the most dangerous.^9^

Central to the diagnosis of BD are the characteristic oral and genital ulcerations and the typical eye involvement associated with the disease. Genetic studies have pinpointed the relevance of HLA‐B51 (B5) carriage, which predominates in males and correlates with certain clinical manifestations, including genital ulcers, ocular and skin manifestations, and a reduced likelihood of gastrointestinal involvement.^9^

### 3.2 BD and spondyloarthropathies

The consideration of BD within the group of negative spondyloarthropathies has been a subject of debate.^10^ While BD can affect sacroiliac joints, the pattern differs from that seen in AS. In BD, there is more common unilateral involvement of sacroiliac joints, contrasting with the bilateral involvement frequently observed in AS.^3^

Research findings indicate that BD does not predispose individuals to a higher prevalence of sacroiliac joint involvement compared to the general population. Therefore, it should not be categorically classified as one of the seronegative spondyloarthropathies.^10^ Moreover, BD exhibits distinct ocular, oral, and genital aphthosis.^2^

### 3.3 Clinical presentation of AS

AS is an inflammatory joint disease primarily affecting the axial skeleton, particularly the sacroiliac joints. While it can present with extra‐articular manifestations, such as eye involvement, the anterior segment of the eye is typically affected, with a relatively favorable prognosis compared to BD.^8^

In AS, extra‐articular manifestations often manifest as anterior eye segment involvement and are associated with a more favorable outcome compared to BD. In some cases, vision loss may occur, but the prognosis is generally more favorable than in BD.^8^

### 3.4 Complex case presentation

Our patient presented a challenging diagnostic scenario, with overlapping clinical features of both AS and BD. The presence of bilateral sacroiliitis at an advanced stage, night pain in the spine, and a positive HLA‐B27 antigen all met the criteria for AS. Simultaneously, the patient exhibited classic features of BD, including oral and genital aphthosis, characteristic ocular involvement, a positive Pathergy test, and a positive HLA‐B51 (B5) antigen.

This case raises questions about the potential overlap or coexistence of these two distinct entities. Similar cases with overlapping presentations have been described in the literature, prompting discussions on whether these conditions represent separate nosological entities or a unique overlap syndrome, akin to mixed connective tissue diseases^4^, ^5^, ^6^

### 3.5 Therapeutic considerations

The management of our patient presented unique challenges due to the dual diagnosis of AS and BD. Conventional immunosuppressive drugs typically used for BD might not be suitable for patients with AS. Additionally, the patient's overweight status posed limitations on the use of TNF‐alpha blockers. The presence of abdominal pain and potential colitis contraindicated the use of IL‐17 blockers. This prompted us to explore alternative therapy approaches, leading us to look deeper into the pathogenesis of BD.

The JAK‐STAT pathway emerges as a pivotal player in the pathophysiology of BD. A comprehensive microarray analysis conducted on BD patients revealed the upregulation of JAK1, particularly in CD14+ monocytes and CD4+T‐lymphocytes, indicating an activated JAK‐STAT signaling pathway.^11^, ^12^ Moreover, the study identified elevated levels of total STAT3 expression in BD, suggesting the involvement of the JAK1/STAT3 pathway. These findings underscore the potential contribution of aberrant JAK‐STAT signaling in the dysregulated immune response characteristic of BD.

In a clinical context, a noteworthy case report detailed the successful use of Tofacitinib (TOF), a small‐molecule pan‐JAK inhibitor, in a refractory BD case with articular, mucocutaneous, and ocular manifestations. The extended‐release formulation of TOF at 11 mg once daily resulted in disease remission, allowing for the cessation of corticosteroid therapy. This therapeutic outcome aligns with the understanding that the JAK/STAT pathway is implicated in BD pathogenesis.^7^ Specifically, TOF's ability to inhibit JAK signifies a promising avenue for treating BD by suppressing the differentiation of pathogenic Th1 and Th17 cells, presenting an alternative to conventional immunosuppressive strategies.

In light of these considerations, we initiated treatment with upadacitinib at a daily dosage of 15 mg. This decision was informed by the reported efficacy of upadacitinib in AS (the only approved JAK inhibitor for AS in our country at the time) and the existence of studies supporting the use of JAK inhibitors in patients with refractory BD.^7^

## 4 CONCLUSIONS

Inflammation of the sacroiliac joints, while a crucial radiographic sign, can be associated with a wide spectrum of diseases, encompassing inflammatory joint conditions, degenerative disorders, trauma, and more. In the case of BD, the possibility of sacroiliac joint involvement has been noted, yet the existing data does not conclusively categorize this disease within the seronegative spondyloarthropathies. Our current patient's dual diagnosis, presenting features of both BD and AS, underscores the pressing need for comprehensive and expansive research efforts.

The choice of treatment for this patient was as intricate as establishing the correct diagnosis. With recent evidence supporting the efficacy of JAK inhibitors in spondyloarthropathies and BD, we opted for a therapy targeting these molecules, which exhibited significant symptom relief. While the coexistence of these two diseases has been reported by various authors over the years, our patient's unique limitations in receiving standard treatments for AS and BD prompted our selection of upadacitinib. Notably, there are no prior reports of upadacitinib usage in BD patients. Nevertheless, upadacitinib has demonstrated its reliability in the treatment of AS, and in our patient, it exhibited notable effectiveness against symptoms typical of BD.

In conclusion, the coexistence of AS and BD within a single patient presents a complex clinical scenario, underscoring the intricacies of diagnosing and managing overlapping rheumatic conditions. This case exemplifies the importance of a multidisciplinary approach to patient care. The initiation of upadacitinib therapy highlights the potential of innovative treatment strategies in addressing the challenges posed by dual diagnoses of rheumatic diseases.

As our comprehension of these conditions continues to advance, further research endeavors and clinical experiences will illuminate the optimal strategies for diagnosis and management. These ongoing efforts hold the promise of enhancing the quality of care provided to patients grappling with complex, overlapping rheumatic disorders.

## AUTHOR CONTRIBUTIONS

**Krasimir Kraev**: Conceptualization; writing—original draft; writing—review and editing. **Petar Uchikov**: Validation; writing—review and editing. **Bozhidar Hristov**: Conceptualization; resources. **Maria Kraeva**: Methodology; writing—review and editing. **Yordanka Basheva‐Kraeva**: Conceptualization; writing—original draft. **Stanislava Popova‐Belova**: Data curation; writing—review and editing. **Milena Sandeva**: Formal analysis; supervision. **Dzhevdet Chakarov**: Supervision; visualization. **Snezhanka Dragusheva**: Conceptualization; formal analysis. **Mariela Geneva‐Popova**: Formal analysis; supervision.

## CONFLICT OF INTEREST STATEMENT

The authors declare no conflict of interest.

## ETHICS STATEMENT

Informed consent was acquired from the patient used in this case report.
